# Simulation study for natural ventilation retrofitting techniques in educational classrooms – A case study

**DOI:** 10.1016/j.heliyon.2020.e05171

**Published:** 2020-10-07

**Authors:** Shouib Nouh Ma'bdeh, Amani Al-Zghoul, Tamer Alradaideh, Asma Bataineh, Saba Ahmad

**Affiliations:** Department of Architecture, Jordan University of Science and Technology, Irbid 22110, Jordan

**Keywords:** Civil engineering, Energy, Environmental science, Energy conservation, Energy use in building, Air quality, Environmental assessment, Green engineering, Architecture, Natural ventilation, Retrofit techniques, Indoor air quality, Indoor air temperature, Energy consumption

## Abstract

Building retrofitting plays a critical role in achieving sustainable development and is an efficient way to improve the indoor air quality (IAQ) of existing spaces. The IAQ in classrooms has a significant impact on the health and academic achievement of students. However, improving the IAQ of existing classrooms is challenging if minimum architectural modifications are allowed. Different natural ventilation retrofitting techniques were proposed to improve the IAQ in existing classrooms at Jordan University of Science and Technology, which is located in a hot arid region. Computer simulations were used to analyze the ventilation rate, indoor operative temperature, relative humidity, and CO_2_ concentration in the base Case classroom and after the implementation of the proposed retrofitting techniques. Simulation results were compared with those obtained in the base case to determine the most efficient natural ventilation retrofitting technique. The best results were obtained by using a solar chimney to assist a wind tower, which resulted in an increase in the comfort hours during the occupation time, an improvement in the average monthly ventilation rate range, a decrease in the CO2 concentration, and an improvement in the relative humidity ratio. An energy-saving of 39% would be achieved compared with the use of split unit air condition systems. Economic assessment of the proposed system using net present value indicates positive economic viability.

## 1 Introduction

Students spend approximately 30% of their student life inside schools, of which 70% of the time is inside classrooms (Wargocki, 2011; Bakó-Biró et al., 2012). Therefore, educational spaces need to be within the comfortable indoor environment quality (IEQ) limits. Furthermore, because educational buildings present a much higher occupancy than other buildings, (1.8–2.4 m^2^/person) compared with offices (10 m^2^/person) (Clements-Croome et al., 2008), and children spend almost 25–30% of their time inside classrooms, and worldwide the length of the education expectancy of children over the age of five has increased from 10.1 years in 1999 to 11.0 years in 2007 (SM, 2018), researchers and designers have paid more attention to issues related to the IEQ in educational spaces. Good IEQ inside educational spaces can directly influence students' outcomes such as satisfaction, productivity, performance, concentration, stress, and learning (Asmar et al., 2014; Choi et al., 2014). On the other hand, poor IEQ in educational buildings could lead to a decrease in general academic performance and achievement, and can result in many illnesses (Mendell et al., 2013; Wu et al., 2019; Wargocki et al., 2020). Classrooms should have a good IEQ, providing: suitable quantities and quality of fresh air, adequate levels of lighting, and thermal comfort (Sarbu and Pacurar, 2015). Many studies have found that the main IEQ factors that affect an occupants’ performance are lighting quality, thermal comfort, and indoor air quality (IAQ) (Sarbu and Pacurar, 2015; Wu et al., 2019).

Worldwide, there are many guidelines and standards references for IEQ and IAQ such as; ASHRAE 55 standard for thermal environment conditions for human occupancy, ASHRAE Standard 62.1 and 62.2 for ventilation for acceptable indoor air quality, REHVA Guidebook 13 for indoor environment and energy efficiency in schools, ISO 7730:2005 for ergonomics of the thermal environment, and CIBSE AM10 for natural ventilation in non-domestic buildings. However, in Jordan, the codes and standards for IAQ that were developed by the Jordan National Building Council are directly based on ASHRAE standards 62 and ASHRAE standards 55, which were used as a reference in this research.

### 1.1 Natural ventilation in classrooms

Natural ventilation is considered to be one of the most used sustainable solutions to ensure healthy and thermally comfortable internal environments, while maintaining lower levels of energy consumption compared to other ventilation strategies (Ji et al., 2009). The potential of applying natural ventilation techniques in classrooms because of its impact on the pupil's health has been raised since the early 1900s (Bakó-Biró et al., 2012). Furthermore, recent studies have addressed the significant role of natural ventilation in improving the indoor environmental conditions in classrooms. Almeida, Pinto et al. (2017) investigated the IAQ in 8 schools and evaluated the potential to improve the IAQ by simple natural ventilation strategies. The results revealed a significant decrease in the CO2 concentration inside the classrooms when using cross ventilation and single-sided ventilation.

Wind towers and solar chimneys are two examples for sustainable solutions used to ventilate the building naturally. Wind towers or wind catchers are natural ventilation elements designed to take advantage of the wind cooling potential. Wind towers can have different shapes and structures and can be located on the roof or next to it, as well as be placed as separate elements connected to the spaces by, for example, an embedded duct (Kleiven, 2003, Kleiven, 2003). Wind towers consist of a vertical shaft with several openings that connect the tower to the rooms to be ventilated. By using the pressure differences surrounding the building, the system provides natural ventilation in the space. The positive pressure on the wind side forces fresh air into the space, and the negative pressure on the leeward side allows stagnant and warm air to circulate. In the absence of wind, buoyancy or the stack effect is the driving force for the wind tower, which is the product of the temperature differential between the micro and macro climates. The resulting difference of the indoor and outdoor air density and pressure gradient allows the warm air (less dense) to rise and escape via the exhaust of the wind tower. Fresh air is then pulled in to replace escaping air (Hughes et al., 2012).

The use of solar chimneys is another sustainable strategy that utilizes solar energy in buildings to improve the efficiency of natural ventilation without relying on the wind (Shi et al., 2018). Its mechanism mainly depends on generating airflow through the conversion of thermal energy into kinetic energy. The provision of the pressure difference between the internal and external space provides the driving force through the creation of a stack effect (Moosavi et al., 2018). The air in a solar chimney gets heated during the day, expands, and rises, in turn pulling the interior air up and out (Chan et al., 2010). The design of solar chimneys consists of three components: outlet and inlet openings, ventilation shaft and the area of the solar collector. The collector is located at the top part of the system. The orientation of the collector, glazing type, thermal, and insulation properties are essential for storing, utilizing, and harnessing the solar gains. The shaft of the system connects the exterior and the interior of the space. Shaft height, thermal properties and cross-sectional area can affect the system performance (Hughes et al., 2012).

### 1.2 IAQ in educational buildings

Health problems caused by indoor air pollution such as sick building syndrome (SBS), have drawn strong concerns (Sun et al., 2015). The IAQ of non-industrial spaces such as educational buildings is an essential determinant of occupant wellbeing and performance (Al Horr, Arif et al., 2016; Kelly and Fussell, 2018). Kats, Perlman, and Jamadagni (2005) and Shaughnessy et al. (2006) have reported that the IAQ and good ventilation in educational spaces may affect health and learning performance. The environmental parameters that affect IAQ or the perceived air quality (PAQ) are: the ventilation rate (VR), indoor relative humidity (RH), air temperature, and gas concentration, such as carbon dioxide (CO_2_), carbon monoxide (CO), nitrogen dioxide (NO_2_), sulphur dioxide (SO_2_), ozone (O_3_) and volatile organic compounds (VOCs) (Yang et al., 2009; Schieweck et al., 2018). The studies proved that the most important factor affecting IAQ is the VR (Seppanen et al., 1999).

#### 1.2.1 Indoor air temperature (iT)

*iT* is an important factor that affects students’ performance (Auliciems et al., 1997). It is one of the main parameters controlled by any ventilation systems in buildings to keep the *iT* from rising above a comfortable level (Linden et al., 2006).

**Figure 1:**
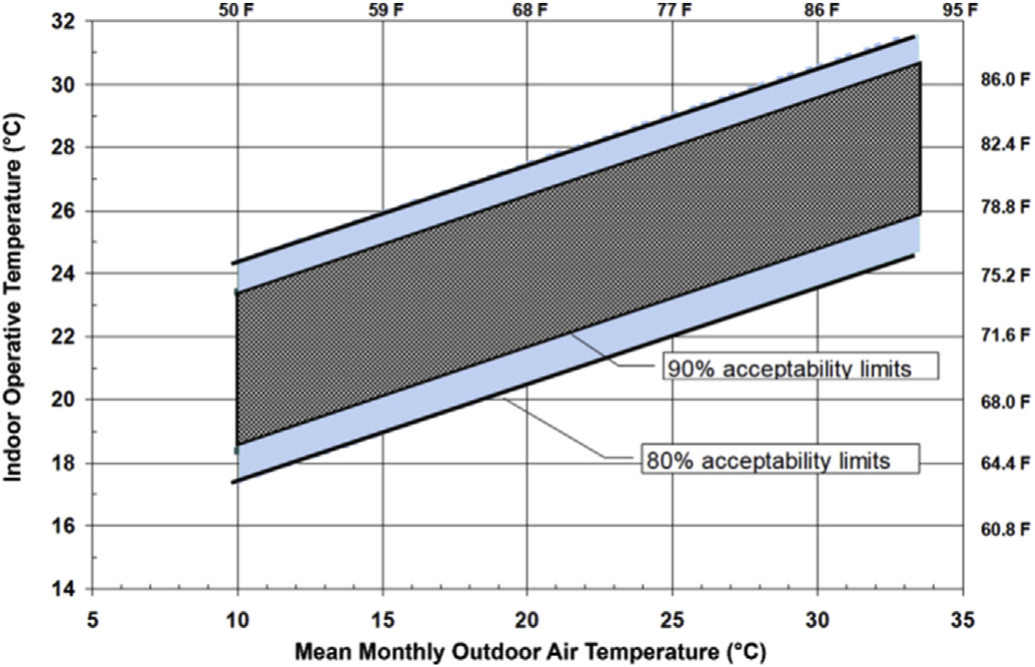
Comfort bandwidths of ASHRAE 55–2017 in naturally conditioned spaces. (ASHRAE) Standard 55 (2017).

The adaptive model of the thermal comfort states that the comfort limits for *iT*'s in naturally ventilated buildings differ from HVAC buildings. It shows that the warm indoor climate in naturally ventilated buildings is acceptable but the same climate is uncomfortable in an indoor air-conditioned building (Dear and Brager, 1998). Studies have also shown that the observed thermal comfort temperatures depend on the outdoor temperature (de Dear and Brager, 2002). The acceptable range of *iT*'s according to the adaptive model of thermal comfort are shown in Figure 1. The ASHRAE 55–2017 was developed for naturally ventilated buildings and mostly depends on the outdoor air temperature based on field measurements.

#### 1.2.2 Ventilation rate (VR)

Educational facilities need ventilation to supply outdoors fresh air to the classrooms to create an acceptable IAQ, which has a positive effect on students’ health and indirectly on the learning performance and achievements (Chenari et al., 2016; Biler et al., 2018). However, the VR in classrooms should be within the required levels to avoid insufficient IAQ and overheating for the occupants (Southall, 2018). The minimum prescribed VR according to ASHRAE Standard 62.1–2019 in a lecture classroom is 4.3 L/s per person.

A study made by Haverinen-Shaughnessy et al. (2011) found a linear relation between classroom VR and students’ academic achievement: “For every unit (1 L/s per person) increase in the ventilation rate within the range of (0.9–7.1 L/s per person), the proportion of students passing a standardized test (i.e., scoring satisfactory or above) is expected to increase by 2.9% (95%CI 0.9–4.8%) for math and 2.7% (0.5–4.9%) for reading”. Clements-Croome et al. (2008) found that “doubling the ventilation rate to 10 L/s per person would improve school performance by 14.5%”.

#### 1.2.3 CO_2_ concentration as an indicator of IAQ and VR

Many researchers have shown that that high CO_2_ concentrations decrease students’ productivity (Shendell et al., 2004; Bakó-Biró et al., 2012; SM, 2018). Coley, Greeves, and Saxby (2007) demonstrated that in a classroom with high CO_2_ levels, students become less interested and attentive, which has a negative effect on learning and educational achievement. Lin and Deng (2003) and Mumovic et al. (2009) have proved that the CO_2_ level in a classroom is a good indicator of the IAQ and the VR. ASHRAE Standard 62.1–2019 states that the level of indoor pollutants such as CO_2_ could be used as an indicator of the VR, and the indoor CO_2_ concentration should be not more than 700 ppm above the CO_2_ concentration of the outdoor air. Liu et al. (2015) reported that an indoor CO_2_ concentration below 800 ppm could decrease SBS. A concentration level exceeding 1,000 ppm is an indication of insufficient ventilation and unacceptable conditions in relation to odor removal (Andamon et al., 2019). The Federation of European Heating, Ventilation, and Air-Conditioning Associations (REHVA) set a performance-based standard that limits the CO_2_ concentration to 1500 ppm over a full school day (Hänninen et al., 2017). Chartered Institution of Building Services Engineers (CIBSE): Application Manual AM10 recommends the CO_2_ concentration to be below 1000 ppm.

#### 1.2.4 Relative humidity (RH)

ASHRAE 62.1–2019 and ASHRAE 55–2017 do not establish lower humidity levels for thermal comfort. However, “comfort factors, such as skin drying, irritation of mucus membranes, dryness of the eyes, and static electricity generation, may place limits on the acceptability of very low humidity environments.” ASHRAE 62.1–2019 recommended that the indoor relative humidity does not to exceed 65%.

RH below 25% is unacceptable because of the effect of dry air on the eyes, skin, and mucous membranes, whereas a RH above 65% may support the growth of pathogenic or allergenic microorganisms. Controlling the level of humidity is very necessary for human comfort. When the *iT* is out of the ASHRAE 55 comfort range, the RH has both indirect and direct effects on comfort and human health. Indirect effects are the impact on pathogenic organisms or chemicals, whereas the direct effects are the effect on physiological processes. The indirect effects are more complex than the direct effect on health (Fang et al., 1998; Bornehag et al., 2001; Woloszyn et al., 2009).

### 1.3 Natural ventilation versus mechanical ventilation

Research results have shown that buildings with mechanical ventilation systems are associated with more SBS symptoms than buildings with natural ventilation systems (Seppanen and Fisk, 2004). Studies have reported that mechanical ventilation systems represent a source of pollution (Bluyssen et al., 1996; Annesi-Maesano et al., 2013). Jomehzadeh et al. (2017) reported that air conditioning systems are associated with an increased level of SBS symptoms, such as headache, dizziness, and throat irritation, in comparison with naturally ventilated buildings (Ji et al., 2009; Suszanowicz, 2018).

Natural ventilation provides a means to control air quality in spaces. It can be used to maintain adequate VR for acceptable IAQ and offers health, productivity, and comfort advantages (Emmerich et al., 2001; Balaban and Puppim de Oliveira, 2017), and it is comfortable for building occupants. Qualitatively, most building occupants prefer natural ventilation systems. According to the study be Emmerich et al. (2001), they stated: “many if not most building occupants may simply prefer natural ventilation systems qualitatively”, and for that reason, architects have accepted and favor the use of natural ventilation and considered it as one of several objectives of high-quality sustainable design. Also, natural ventilation offers users more freedom in control of their environments and the ability to adapt their environment to their immediate perception of comfort. Another study by Gunnarsen (2000) of a school in which the mechanical system was replaced by a natural ventilation showed that: “the school users were as good, or better, at achieving a comfortable temperature and air quality as the poorly maintained mechanical ventilation system with central automation” (Aizlewood and Dimitroulopoulou, 2006; Porteous, 2019; Izadyar et al., 2020).

Natural ventilation can provide fresh air for occupants, cool buildings, and maintain an acceptable level of air quality when the outdoor climatic conditions allow (Emmerich et al., 2001; Heiselberg, 2004). The larger turbulence intensity of natural wind enhances the feeling of comfort because it intensifies the heat convection between people and the environment (Su et al., 2009). Air movement in mechanical ventilation is associated with noise, whereas air movement in natural ventilation is silent, associated with a quiet environment (Kleiven, 2003, Kleiven, 2003; Gratia et al., 2004). On the other hand, the dependency of natural ventilation on the available climatic conditions (ambient temperature and humidity, air pollution, and acoustic pollution) and the characteristics of the building are its two major drawbacks (Causone, 2016; Tan and Deng, 2017).

Despite the widespread use of mechanical ventilation systems, because of the drawbacks and negative effect of mechanical ventilation systems on both the occupants and the environment, architects and engineers have returned to using sustainable, clean technology and energy-efficient natural ventilation systems (Linden et al., 2006; Chenari et al., 2016). Mechanical ventilation systems contribute to a significant portion of the total building energy consumption among all building services (Liddament, 2000; Pérez-Lombard et al., 2008). Balaban and Puppim de Oliveira (2017) reported that the use of innovative ventilation systems instead of mechanical ventilation could reduce energy consumption.

In this research, different natural ventilation retrofitting techniques were investigated to improve the IAQ in existing classrooms. The performance of these techniques was evaluated in terms of *iT*, ventilation rates (VR), CO_2_ levels, and RH. The suggested techniques were tested using a typical classroom at Jordan University of Science and Technology (JUST), Jordan, as a model in computer simulations. The suggested techniques were selected to have a minimal modification to the building envelope owing to the special construction system used in the building—precast concrete with bearing load. They can be fixed with minimal modifications to the building envelope and layouts and have a minimal impact on the building's spatial design. Also, the installation of the suggested modifications will help to reduce the University's total energy consumption and the consequential environmental effects of using a newly installed air conditioning systems. Energy and economic assessment were performed to assess the viability of the proposed systems.

### 1.4 Case study

**Figure 2:**
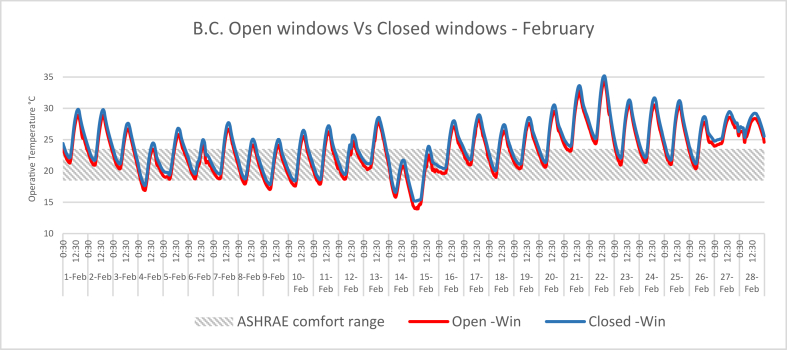
Indoor air temperatures in the B.C during February when the windows are open versus closed.

**Figure 3:**
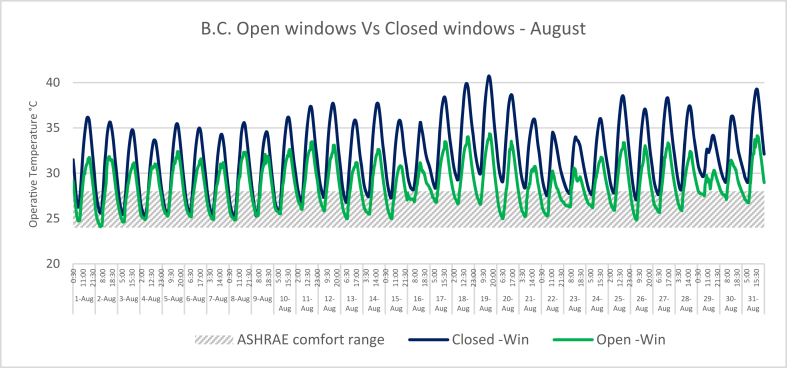
Indoor air temperatures in the B.C during August when the windows are open versus closed.

In this research, the computer simulation results of the IAQ for a typical classroom located at JUST are presented. The University was built in 1986 and is located in Jordan (32°33′0″N and 35°51′0″E). The climate of the campus is hot arid, which means that it is dry-hot in the summer and wet-cold in the winter. Buildings in hot arid desert climates can benefit from natural ventilation through the stack effect, to draw air through evaporative cooling systems or by wind pressure at night to enhance cooling of the building during the night (Badran, 2003). The construction system used in the University's buildings is a prefabricated mass concrete system, which can be used for nocturnal convective cooling. However, opening the classroom windows is not sufficient to bring the classroom environment to the comfort zone during the operation hours, as shown in Figure 2 and Figure 3.

In addition to the low comfort hours, and owing to noise issues coming from the adjacent courtyards, the classrooms’ occupants tend to close the windows and thus impede the cross-ventilation process. For many reasons, the solution from the building administration office was to install new split-unit air conditioning systems to adjust the thermal environment of the classrooms to be within the comfort zone. This increased the total energy demand but did not allow the control of other IAQ variables such as odors and CO_2_ levels.

**Figure 4:**
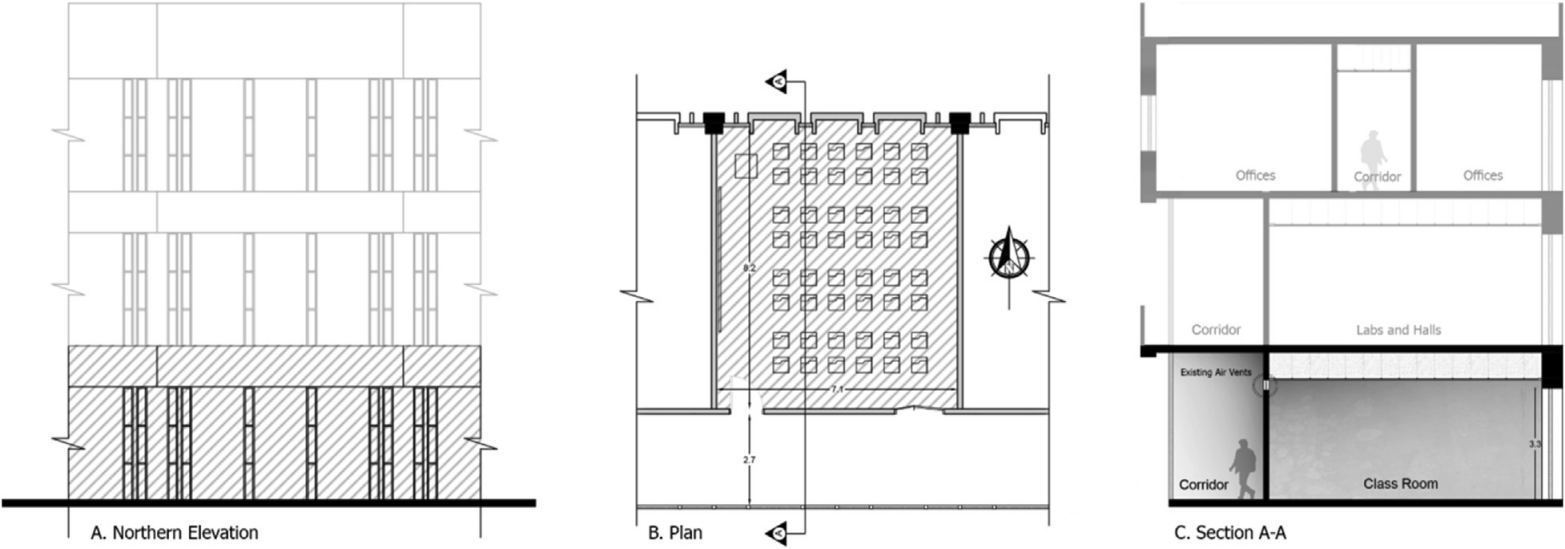
Elevation, plan, and section of the B.C.

**Figure 5:**
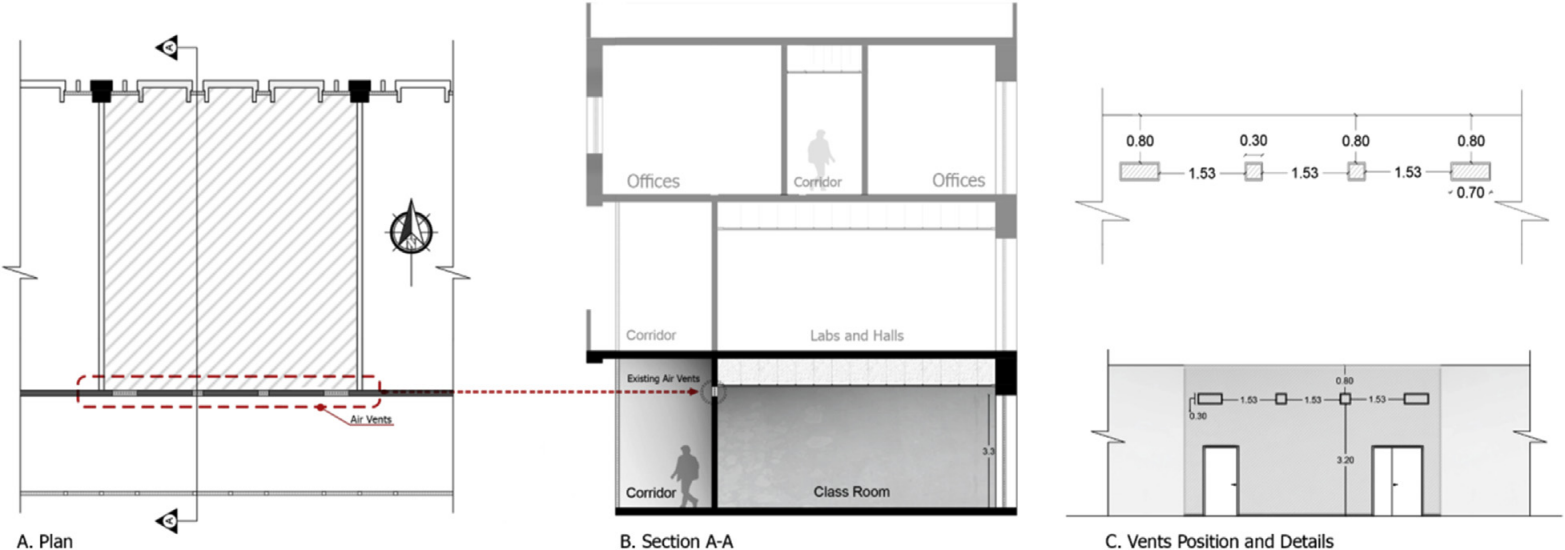
Plan and section of a typical classroom showing the position and dimensions of the internal vents.

The characteristics of the simulated classroom that was used in this study are the following: the simulated classroom is located on the ground level with single-loaded corridors, and represent approximately 60% of the 152 classrooms at JUST. The classroom is oriented toward the north. The classroom dimensions were 7.1 × 8.2 × 3.5 m (length × width × height), with a volume of 203.7 m^3^, and 0.80 m false ceiling cavity, as shown in Figure 4. The single-loaded corridor had a width of 2.7 m at the southern side of the classroom. The external windows of the classroom were facing north, 0.3 m wide and 3.3 m high, with a total window-to-wall ratio of 19%. 30% of the total windows’ area was operable. The spatial characteristics such as dimensions and capacity were found to be similar to other studies in the literature that investigated the potential of natural ventilation in educational buildings (Jones and Kirby, 2012; Wang et al., 2014). The classroom had four small windows (vents) at the top of the southern wall of the classroom (internal partition between the classroom and the corridor) to improve the air movement inside the classroom—cross ventilation. The dimensions and locations are illustrated in Figure 5.

The typical capacity of the classroom is 48 students with an occupant density of 1.2 m^2^/occupant. The thermal transmittance (U values) of the classroom envelope are as shown in Table 1, which comply with the Jordanian national building code. The low U values of the building elements shows that it was designed with consideration to the hot arid climate of the building's location. The university operation schedule is presented in Table 2. The University occupancy period is five days (Sunday -Thursday) form 8:00 AM–6:00 PM. Architectural drawings for the base Case (B.C) study were collected from the Engineering Projects Unit at the University.

**Table 1:** Materials’ and U-value of classroom envelope.

Member	Material, thickness	U-value (w/m2-k)
Ceiling	Suspended ceiling, Ceiling tiles, Thickness: 0.020 m	1.7
Internal partition	Brick, Thickness: 0.100 m Plasterboard, Thickness: 0.012 m	1.6
External Walls	Concrete, Thickness: 0.070 m Expanded polystyrene, Thickness: 0.050 m Concrete, Thickness: 0.100 m	0.4
Sliding Window	6 mm double pane of glass with 12 mm air gap and 0.03 m aluminum frame	1.6

**Table 2:** University operating schedule.

	Period	Occupied spaces
Fall semester	Mid September – Mid January	100%
Spring Semester	Early February – Late May	100%
Summer semester	Late June – Early September	90%

## 2 Material and methods

**Figure 6:**
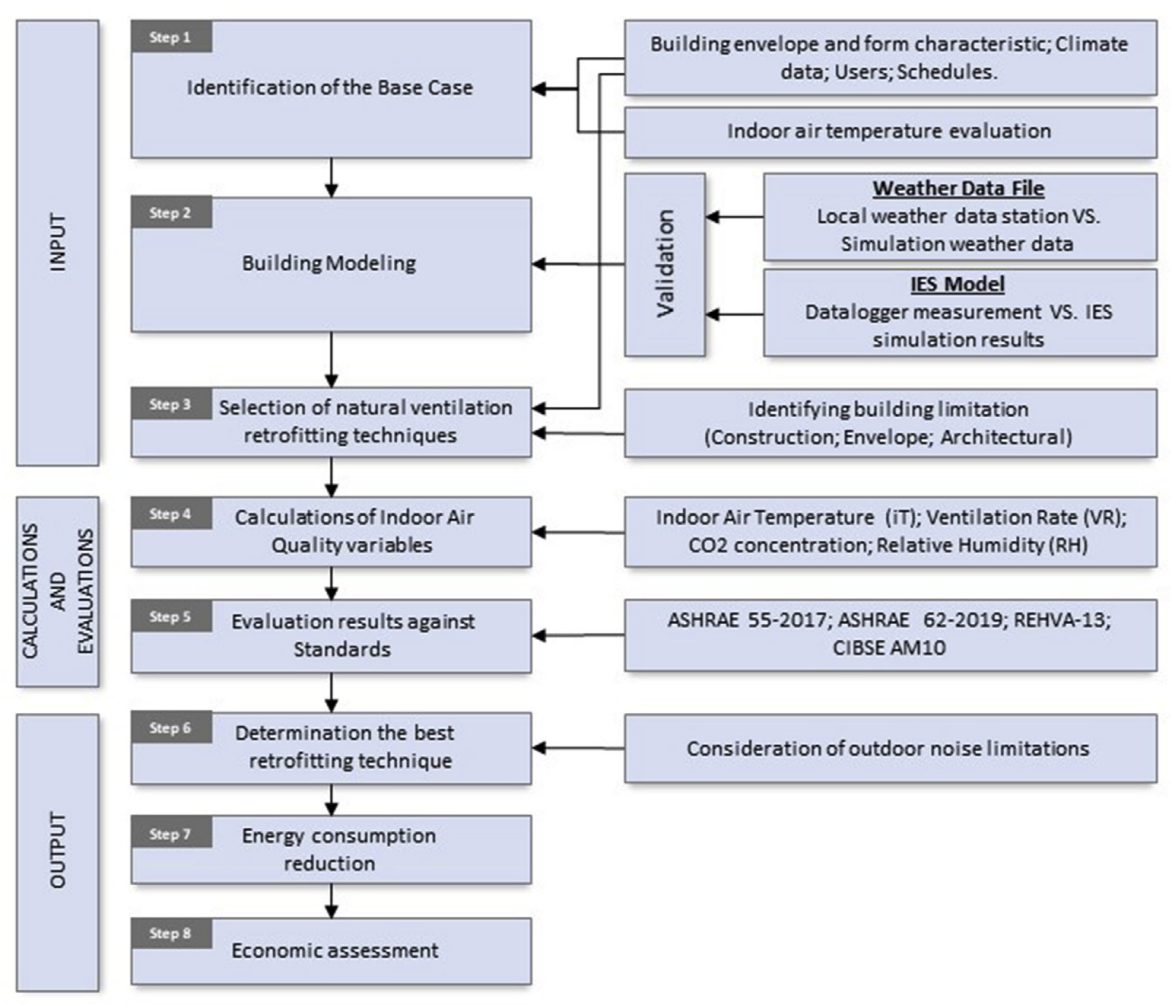
Research methodology for selecting the best natural ventilation retrofitting techniques.

The research method that was followed to select the best natural ventilation retrofitting techniques to improve the IAQ in existing classrooms, is explained in Figure 6. These steps could be adapted to investigate other passive retrofitting techniques.

Step 1: Identifying the B.C, including the building envelope and form, the climatic data, users and their schedule, and preliminary evaluation of the indoor air temperature. This step is explained in the previous section.

Step 2: Modeling of the building using computer simulation software and validating the results of the simulation and the weather data used by the software.

Step 3: Selecting the appropriate natural ventilation retrofitting techniques, taking into consideration the results of step 1.

Step 4: Calculating the IAQ variables related to the implementation of the proposed systems indicated in step 3.

Step 5: Evaluating the results of step 4 against relevant standards.

Step 6: Determining the best natural retrofitting techniques considering any limitations preventing the applicability of the proposed systems, e.g., noise.

Step 7: Calculating the energy reduction associated with the implementation of the proposed system to reinforce any related decision.

Step 8: Performing an economic assessment for the selected systems to determine the cost of the investment.

### 2.1 Building modeling

Computer simulation was used to conduct environmental properties for the B.C and the proposed natural ventilation retrofitting techniques. Apache-sim, a plug-in to IES-VE software, was used to build the model for the simulations, and it was used to simulate the classroom thermal condition and the airflow.

IES-VE was used to investigate the proposed natural ventilation retrofitting techniques IES-VE is a user-friendly, well-established simulation tool that analyzes the performance of different building systems and allows their optimization considering comfort and energy (IES, 2017). It has been widely used among researchers since its methodology of calculations has been widely validated, meeting the requirements of various regional and international standards, such as the American Society of Heating, Refrigerating and Air Conditioning Engineers (ASHRAE) Standard 140, USGBC (LEED Automation Partner), UK National Calculation Methodology (NCM), ASHRAE 55 calculation procedure, and ISO 7730 calculation procedure (IES, 2017). This software provides researchers with various simulation packages; however, herein, only three of them were used, which are ModelIT for modeling the B.C unit and proposed modifications, ApacheSim for thermal analysis, and Macroflo for natural ventilation analysis.

#### 2.1.1 Validating the weather data file

The data of outdoor temperature, wind speed, and direction were obtained from the software weather data file. Average monthly mean dry bulb (DB) temperatures for the university location are listed in Table 3, along with the average monthly DB temperature obtained from the simulation program weather file. To validate the simulation weather data, the two values were compared. A high agreement between the values was obtained, with a discrepancy range of 2–6% owing to the variability of the outdoor conditions. However, as the weather file obtained from the local station lacked some critical information such as hourly wind speed, wind direction, and solar radiation, the weather data from the simulation software were used in this study.

**Table 3:** Average monthly outside DB temperatures, Irbid, Jordan.

Date	Average Monthly Outside DB Temperatures		
	Simulation program Dry bulb	Irbid Weather Station	Discrepancies
1/1/2019	8.2	8.7	6%
2/1/2019	11.8	12.4	5%
3/1/2019	12.1	12.7	5%
4/1/2019	16.4	17.1	4%
5/1/2019	21.9	22.5	3%
6/1/2019	24.0	24.5	2%
7/1/2019	26.9	27.5	2%
8/1/2019	26.0	26.6	2%
9/1/2019	23.5	23.9	2%
10/1/2019	20.3	21.0	3%
11/1/2019	14.6	15.0	2%
12/1/2019	10.3	10.7	4%

According to the Jordan Ministry of Environment, the Air Quality Index (AQI) of Jordan is classified as Good to Moderate. This classification is based on the U.S EPA AQI Standards. For the location in this study, historical data shows that the particulate matter 10 μm (PM10) levels ranged between 10-93 μg/m^3^, nitrogen dioxide (NO2) 5–15 ppm, and carbon monoxide (CO) 11–33 ppm (Jordan Ministry of Environment, 2020).

#### 2.1.2 Validating the IES model

For accurate results, the simulation software was set to perform annual energy, thermal, natural ventilation, and CO_2_ simulation with 2 steps per hour. The simulation software produces energy and comfort analysis for the building during the selected months. However, running a continuous simulation in 30-minute increments provides the proposed systems the time required for full operation. In addition, during this time, the software considers the loss of efficiency that occurs overnight or the overheating during some days. Therefore, the results presented in this research were selected to illustrate the effectiveness of the proposed systems during the selected months of cooling and heating seasons in detail.

The simulation results showed the iT's, VR, CO_2_ concentration, and RH of the indoor space, during the usual hours that the classroom was occupied (8.00 AM- 6.00 PM). The indoor air temperatures of the B.C model were validated using site measurements to validate the simulation results. The indoor air temperatures were recorded during the occupation period for one typical week during the summer season (August) and one typical week during the winter season (February). One data logger was installed in the sample classroom. Extech SD800 datalogger was installed in the classroom, the data logger was used to measure the indoor air temperature, RH, and CO_2_ concentration with an accuracy of 0.8 °C, ±4%, and ±40 ppm respectively. The datalogger was mounted 1.2 m above the floor in the middle of the classroom, and the measurements were recorded at 15-min intervals when the occupancy of the classroom is ≥ 80%. There was an agreement between the results obtained from the simulation program and the measured values, with an average error of 2–3% during the whole week.

According to the adaptive model indicated in Figure 1, and based on the weather data of the Case study, the acceptable iTs ranges for the selected months; February and August are as shown in Table 4.

**Table 4:** Acceptable indoor air temperatures according to ASHARE adaptive model.

Month	Mean monthly outdoor air temperature	Comfort range for indoor operative temperature
February	11.87 °C	18.5–23.5 °C
August	26.07 °C	24–28 °C

### 2.2 Proposed natural ventilation retrofitting techniques

Several natural ventilations retrofit techniques were proposed to improve the IAQ of the B.C.

**Figure 7:**
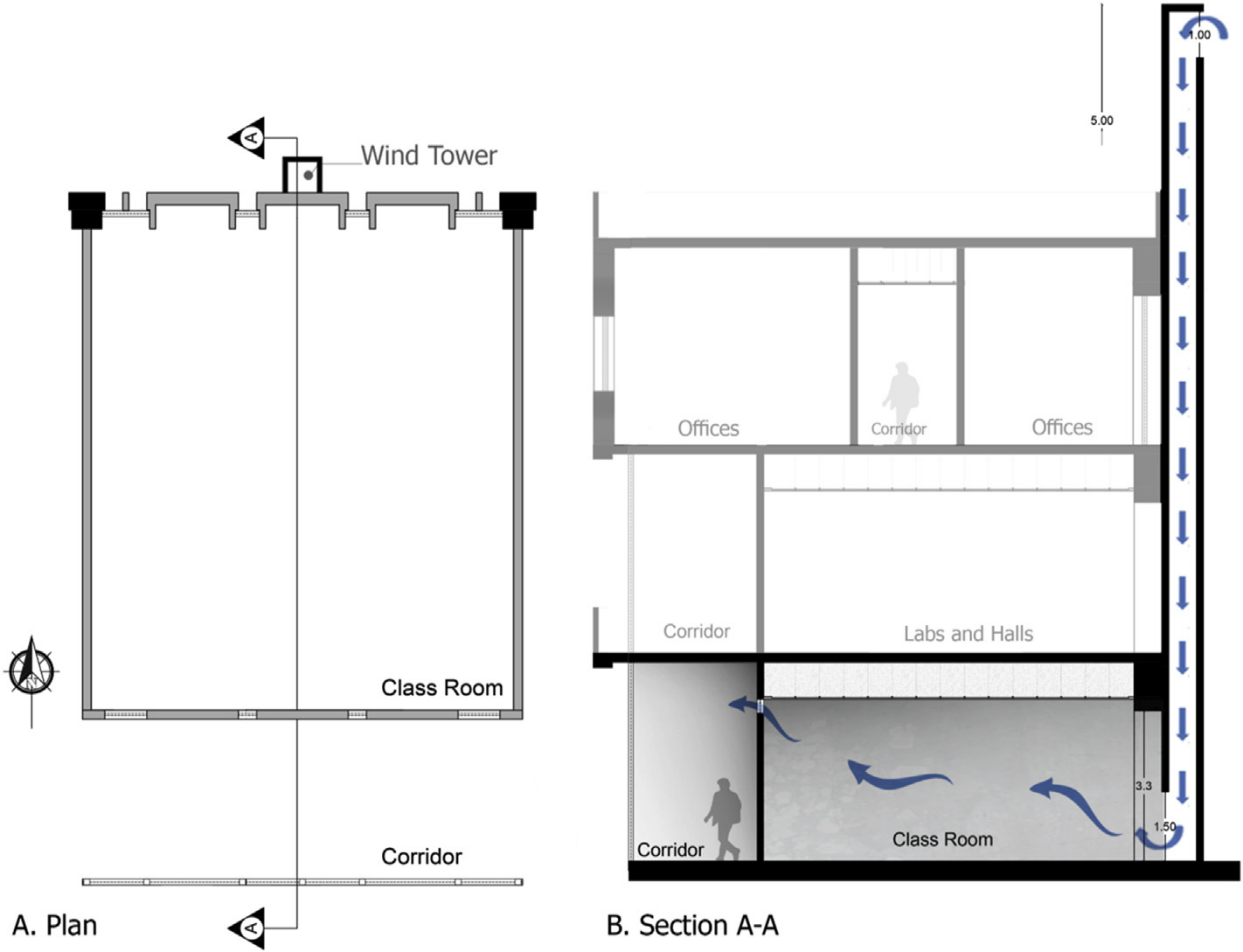
Plan and section of a typical classroom showing the suggested wind tower position and dimensions.

**Figure 8:**
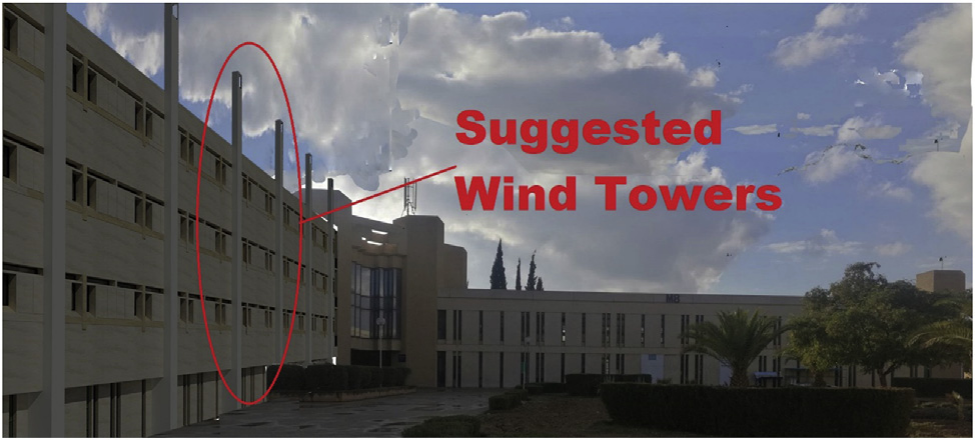
Illustration photo for the appearance of the suggested wind towers.

Case No.1 One wind tower installed on the northern side of the classroom (adjacent to the external wall) at the middle of the classroom, and the original windows of the classroom are closed. The wind tower characteristics used by Badran, (2003) were used in this study. The tower had a height of 5 m from the top of the last floor and a 0.57 × 0.57 m cross-section. The tower had two top openings on the northeast elevation to catch the prevailing wind, and one opening at the bottom towards the internal space of the classroom to let air in, as shown in Figure 7 (see Figure 8).

Case No.2 One wind tower installed on the northern side of the classroom, as in Case No.1, with 30% of the original external northern windows' area open to allow nocturnal convective cooling during nights when the outdoor DB temperatures are suitable.

**Figure 9:**
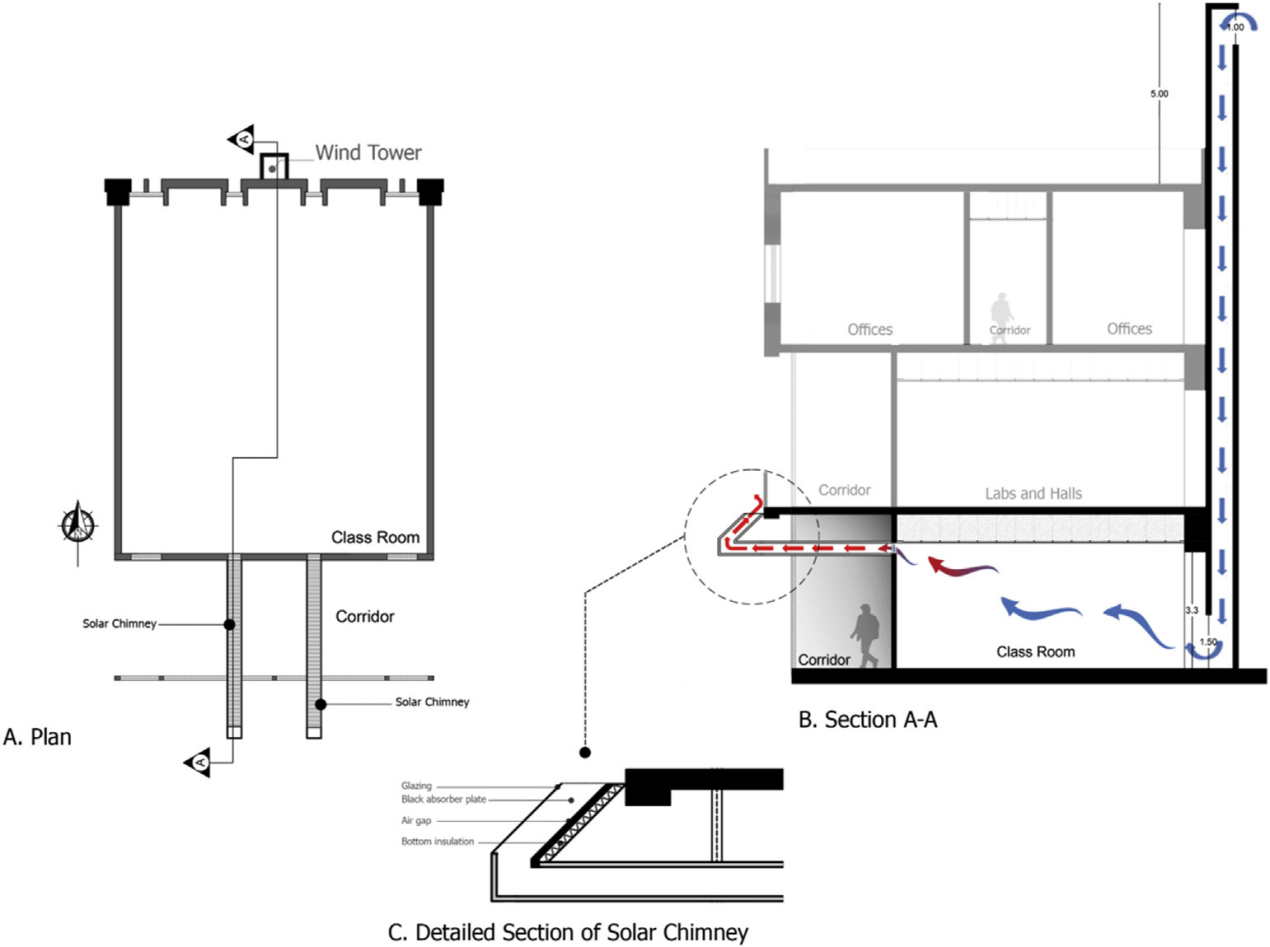
Plan and section of a typical classroom showing the suggested solar chimneys and the wind tower positions and dimensions.

**Figure 10:**
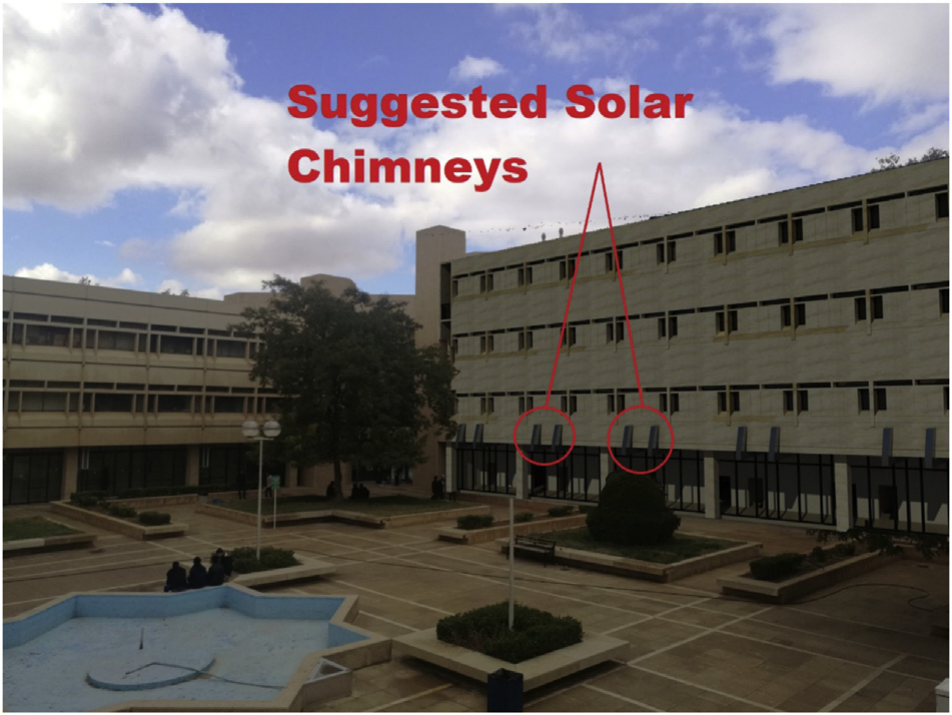
Illustration photo of the appearance of the suggested solar chimney.

Case No.3 In this case, two solar chimneys on the southern side of the B.C were added and replaced the two small vents in the middle at the common wall (Figure 5c). These solar chimneys were installed in addition to the wind tower described in Case No.1. The purpose of the solar chimneys was to help extract the hot air from the classroom and let in more cool air through the wind tower. The horizontal part of the solar chimney was made from aluminum, extended 3.15 m outside the southern windows along the corridor. The solar chimney was 1.50 m high and inclined by 45° and had a cross section of 0.30 × 0.30 m, as shown in Figure 9. Table 5 shows the material components of the two systems. The cross-section of the solar chimney was determined (fixed) based on the existing vent size at the common wall. The height of the solar chimney was chosen so that it would have minimal interference with the architectural appearance of the façade. However, even though the proposed solar chimney design was not optimal because of these limitations, the solar chimney dimensions, the ratio between inlet and outlet area, and height/gap ratio do not conflict with findings reported in many other studies (Bouchair, 1994; Chen and Li, 2001; Chen et al., 2003; Shi et al., 2018). The inclination angle was determined based on the location latitude, as indicated in a study by Mathur et al. (2006) (see Figure 10).

**Table 5:** Material's and U-value of wind tower and solar chimney.

Element	Physical characteristics		
	Member	material and Thickness	U-Value w/m2-k
Wind tower	walls	Cultivated Peat Soil 133%D.W. Moisture, thickness 0.10 m	2.00
Solar chimney	Horizontal Duct	Aluminum, 0.005 m	7.3
	Diagonal Part	clear float 6mm, Thickness: 0.006 m	0.54
		Black absorber, 0.05 m	
		Air gap, 0.20 m	
		Expanded polystyrene, 0.05 m

Case No.4 The same parameters as Case No.3 were used, except 30% of the original external northern windows' area were opened to allow nocturnal convective cooling by letting more cool air inside the space when the outdoor dry bulb temperatures are suitable.

### 2.3 Economic life cycle assessment

In most cases, sustainable decisions are based on social and environmental factors. Improving the natural ventilation in classrooms also has many qualitative benefits. However, economic factors are one of the most important driving forces of investment in energy retrofitting systems. Therefore, we analyzed the quantitative data of the economic life cycle assessment of implementing the suggested system. We performed an economic life cycle assessment (LCA) using the method described in (Randolph and Masters, 2018).

#### 2.3.1 Simple payback period (SPP)

SPP is a static method that can be used to calculate the number of years it will take for the initial cost of a system to be recouped based on energy saving, using the following equation (Eq. 1):

(1) SPP=IC/(AES∗Pr)

in which: *IC* is the initial capital cost in Jordanian Dinar (JD), *AES* is the annual energy savings (kWh/yr), and *Pr* is the energy price (JD/kWh).

#### 2.3.2 Net present value over life cycle

There are many limitations when using SPP over a long time period and high discount rates, as it ignores the time value of the money and the operation and maintenance cost. However, despite these limitations, it is still a useful economic measure for cost effectiveness. To address the limitations, dynamic methods such as net present value (NPV) are preferred for calculating the economic life cycle cost as the present value life cycle assessment is used. NPV is a standard method to calculates the total money savings of the energy investment in present-day money. A positive NPV value indicates a good investment and an appropriate choice for the retrofitting option (Eq. 2).

(2) NPV=PVS−IC

in which: *PVS:* is the present value savings (Eq. 3):

(3) PVS=(AES∗Pr−O&M)∗UPVF

in which: *O&M* is the annual operation and maintenance cost (JD) and *UPVF:* is the uniform present value factor (Eq. 4):

(4) UPVF=(((1+d)ˆn−1))/(d〖(1+d)〗ˆn)

in which: *d* is the discount rate.

#### 2.3.3 Benefit-cost ratio

The benefit-cost ratio (B/C) compares the annualized money savings and the annualized cost of the system to provide a ratio of benefits to cost (Eq. 5). A larger ratio indicates a more cost-effective investment.

(5) B/C=PVS/IC

## 3 Results and discussion

### 3.1 Indoor air temperature (iT)

#### 3.1.1 Heating season: February

**Figure 11:**
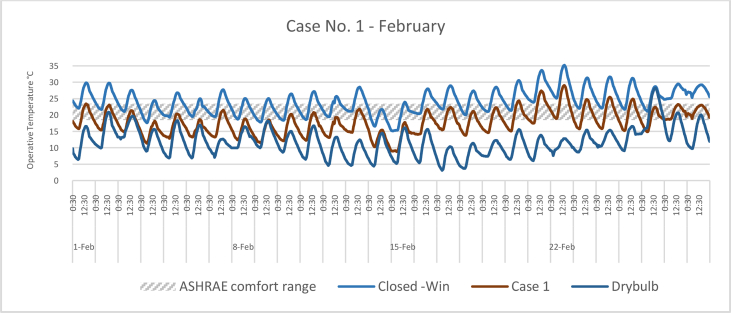
*iT*s for the B.C and Case No.1 compared with outdoor DB temperatures; February.

**Figure 12:**
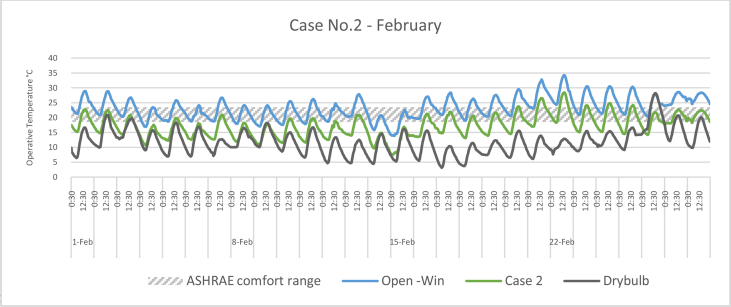
*iT*s for the B.C and Case No.2 compared with outdoor DB temperatures; February.

**Figure 13:**
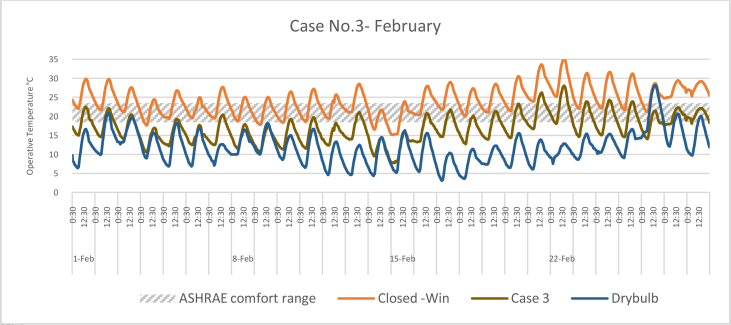
*iT*s for the B.C and Case No.3 compared with outdoor DB temperatures; February.

**Figure 14:**
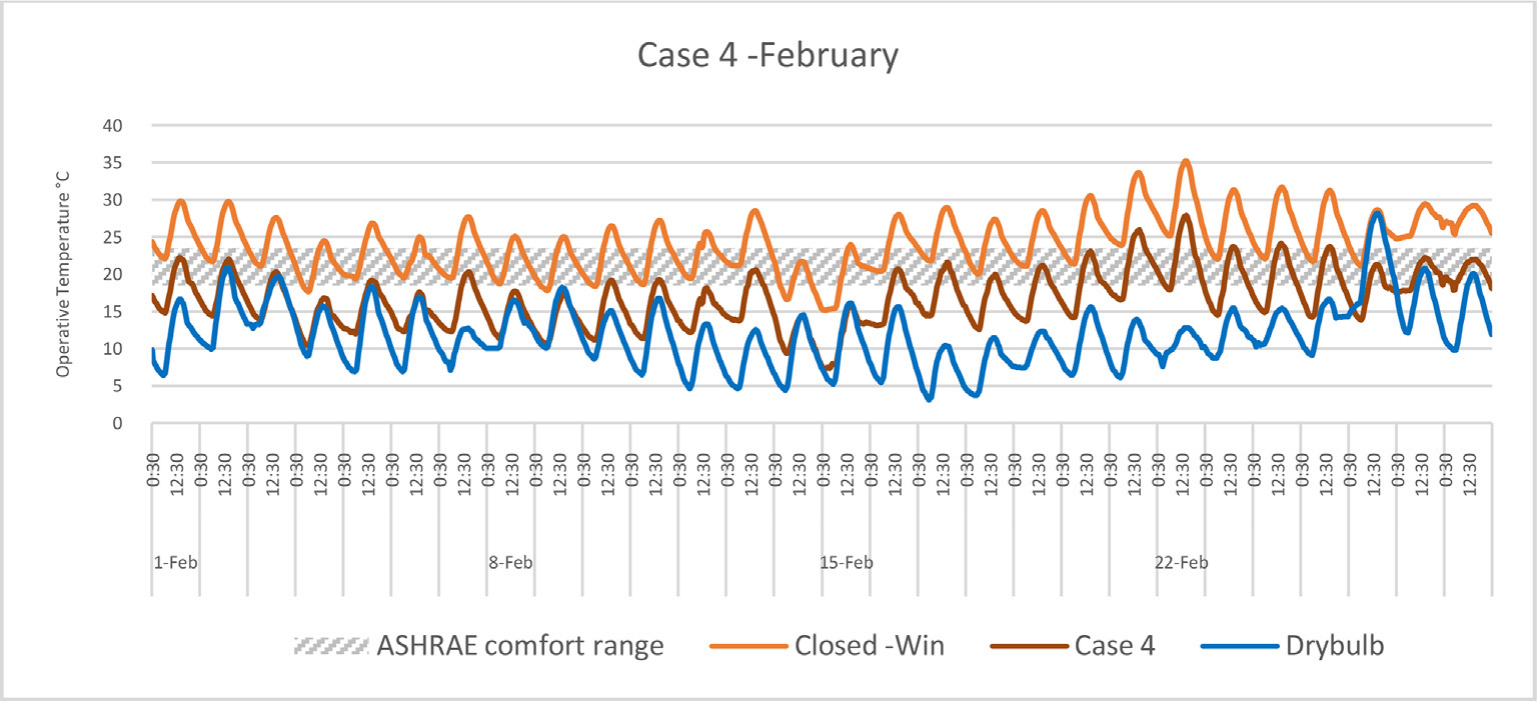
*iT*s for the B.C and Case No.4 compared with outdoor DB temperatures; February.

**Figure 15:**
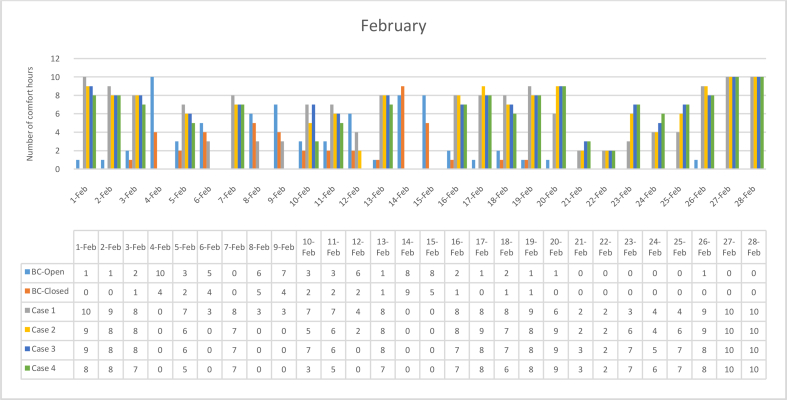
Daily comparison of comfort hours during occupation hours for the B.C and the simulated techniques; February.

The *iT*s during February for each Case compared with the B.C are shown in Figures 11, 12, 13, and 14. To measure the effectiveness of each system, the number of hours during which the iTs are within the comfort zone according to the ASHRAE adaptive model as presented in Section 1.3, during the occupation time, were calculated and presented in Table 6 and Figure 15. The case that resulted in the maximum number of hours during which iTs were within the comfort zone range was case No.1, followed by Case No.3, Case No. 2, and Case No.4.

**Table 6:** Number of comfort hours during occupation hours for the B.C and the simulated techniques; February.

February comfort hours (90% acceptable limits; ASHRAE 55–2017)						
Day	B.C-Open	B.C-Closed	Case 1	Case 2	Case 3	Case 4
1-Feb	1	0	10	9	9	8
2-Feb	1	0	9	8	8	8
3-Feb	2	1	8	8	8	7
4-Feb	10	4	0	0	0	0
5-Feb	3	2	7	6	6	5
6-Feb	5	4	3	0	0	0
7-Feb	0	0	8	7	7	7
8-Feb	6	5	3	0	0	0
9-Feb	7	4	3	0	0	0
10-Feb	3	2	7	5	7	3
11-Feb	3	2	7	6	6	5
12-Feb	6	2	4	2	0	0
13-Feb	1	1	8	8	8	7
14-Feb	8	9	0	0	0	0
15-Feb	8	5	0	0	0	0
16-Feb	2	1	8	8	7	7
17-Feb	1	0	8	9	8	8
18-Feb	2	1	8	7	7	6
19-Feb	1	1	9	8	8	8
20-Feb	1	0	6	9	9	9
21-Feb	0	0	2	2	3	3
22-Feb	0	0	2	2	2	2
23-Feb	0	0	3	6	7	7
24-Feb	0	0	4	4	5	6
25-Feb	0	0	4	6	7	7
26-Feb	1	0	9	9	8	8
27-Feb	0	0	10	10	10	10
28-Feb	0	0	10	10	10	10
No. comfort Hours	72	44	160	149	150	141
Total Hours = 280 (8:00 AM - 6:00 PM)	26%	16%	57%	53%	54%	50%

The wind tower harnesses and utilizes the prevailing wind to cool and circulate air through the space. The driving force for the wind tower is buoyancy or the stack effect, which is a result of the *iT* difference between the interior and exterior climate. The subsequent variation in air density and pressure gradient of the indoor and outdoor air masses causes the warm air—less dense—to rise up and escape through the exhaust, whereas the solar chimney extracts the hot air from the space at the same time. Consequently, new air is drawn in to replace the air that has escaped. When a solar chimney is in operation, it helps with cross ventilation and air movement and works as a shaft for the hot air exhaust from the space, and decreasing the *iTs*. On a limited number of days, when the outdoor air temperatures were high, the proposed systems were not enough to decrease the *iTs* to be within the comfort limits.

In all cases, the number of hours during which the iTs fall within the comfort zone significantly increased. The proposed systems maximize the air movement in the space and improve the cross-ventilation. Thus, *iT's* were decreased below what was registered in B.C. When comparing Case No.1 and Case No.3 with Case No.2 and Case No.4, respectively, it is evident that the introduction of more outdoor air with low DB temperatures (Case No.2, and Case No.4) resulted in a decrease in the iTs below the comfort zone indicated by ASHRAE 55–2017, thus reducing the number of comfort hours. The use of a solar chimney, which helps in increasing the cross-ventilation, resulted in more air with low DB temperatures and thus lowering of the number of comfort hours.

#### 3.1.2 Cooling season: August

**Figure 16:**
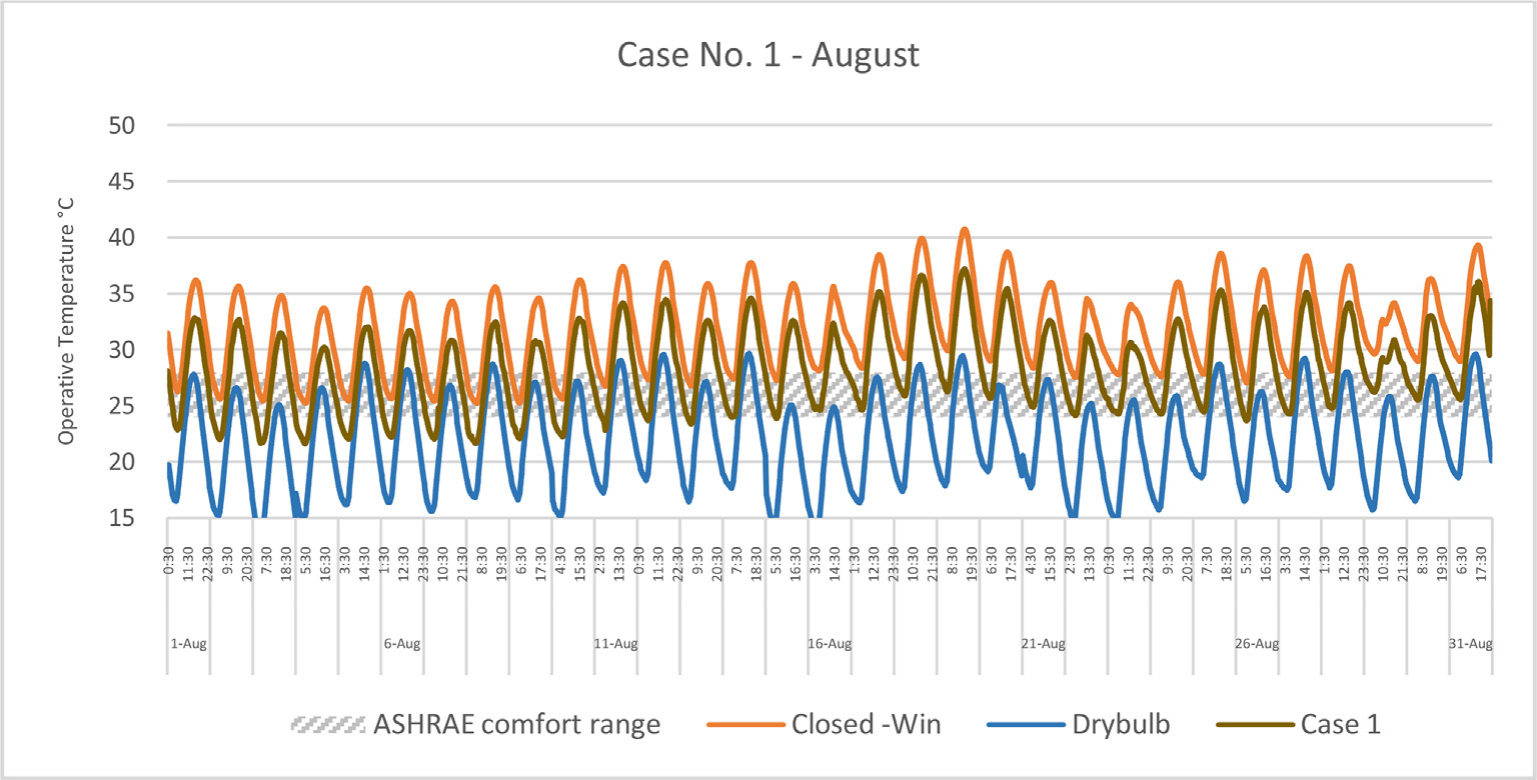
iTs for the B.C and Case No.1 compared with outdoor DB temperatures; August.

**Figure 17:**
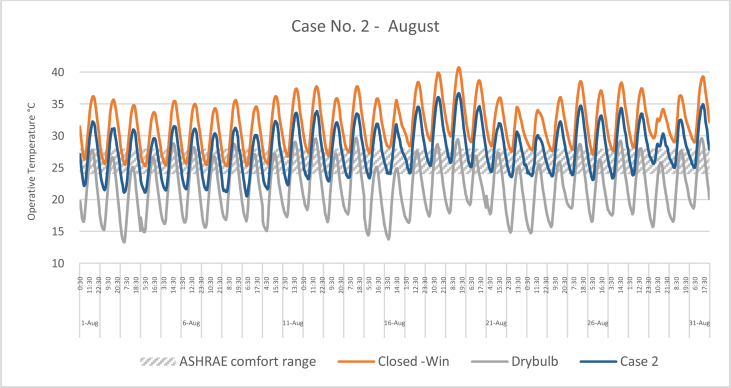
iTs for the B.C and Case No.2 compared with outdoor DB temperatures; August.

**Figure 18:**
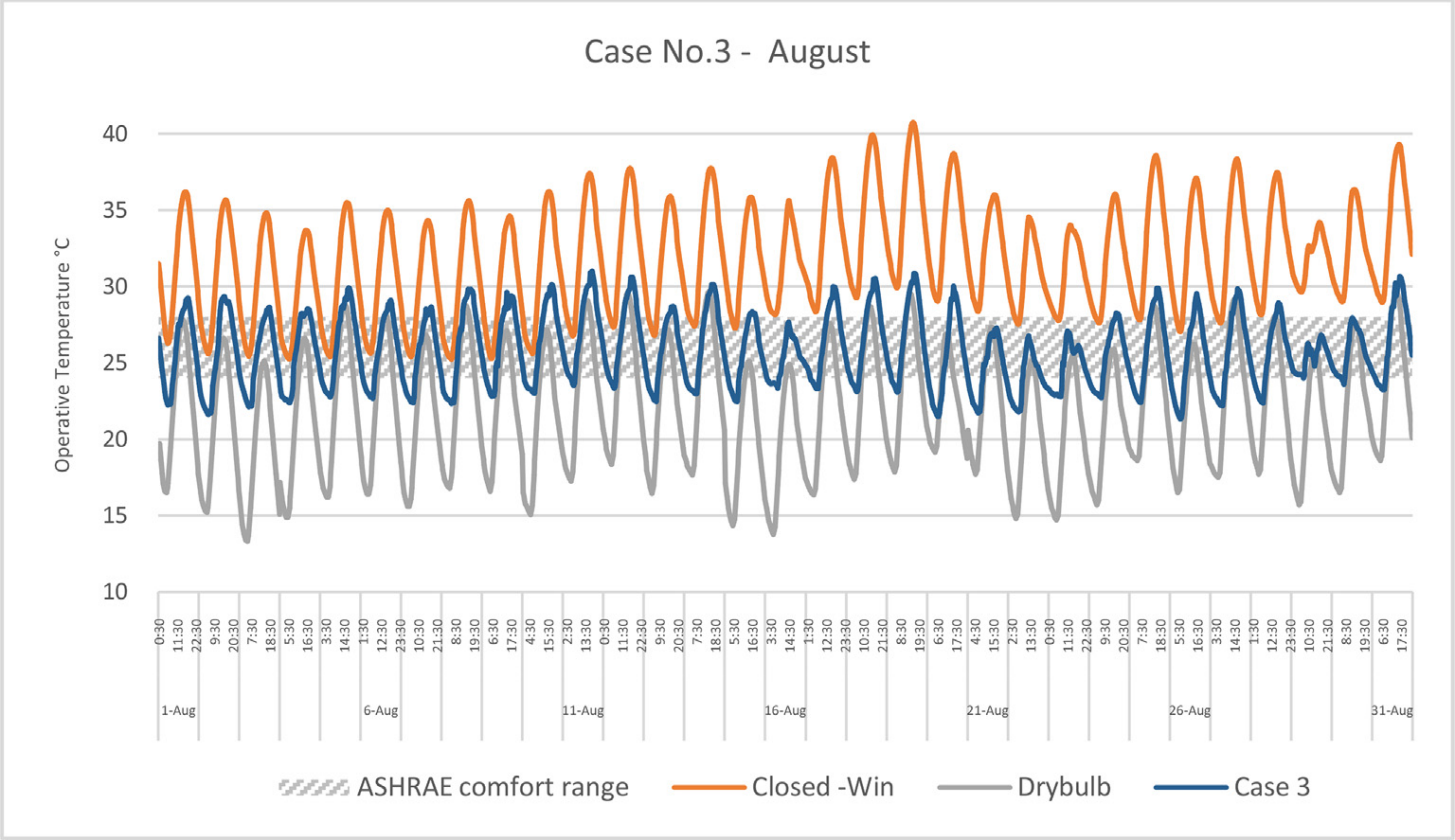
iTs for the B.C and Case No.3 compared with outdoor DB temperatures; August.

**Figure 19:**
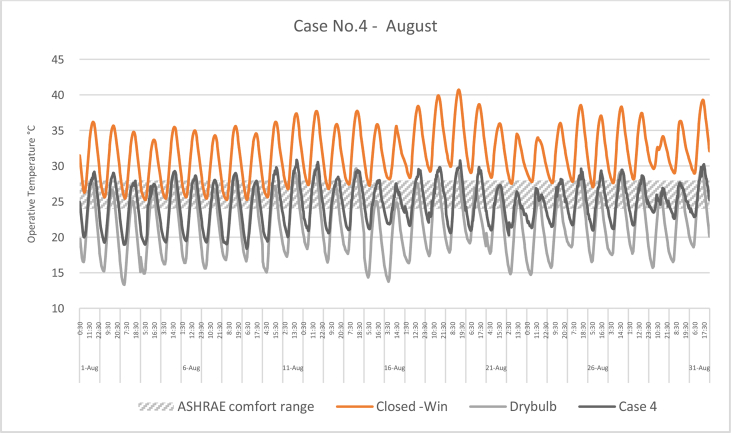
iTs for the B.C and Case No.4 compared with outdoor DB temperatures; August.

**Figure 20:**
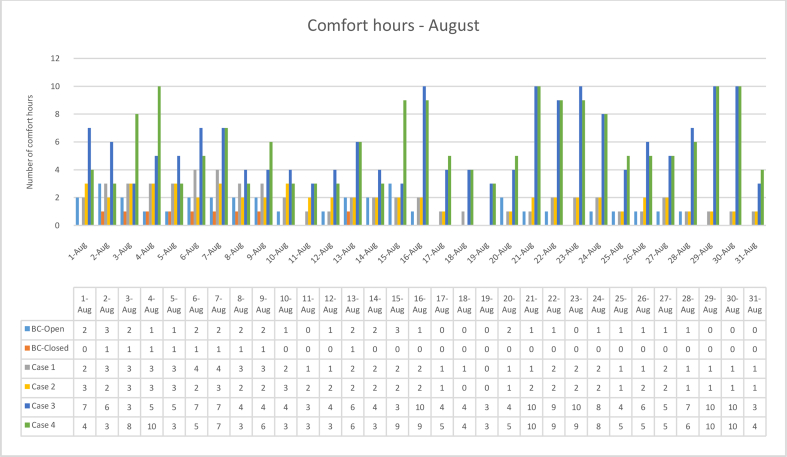
Daily comparison of comfort hours during occupation hours for the B.C and the simulated techniques; August.

The iTs during August for each Case compared with the B.C are shown in Figures 16, 17, 18, and 19. To measure the effectiveness of each system, the number of hours during which the iTs were within the comfort zone according to ASHRAE adaptive model, as presented in Section 1.3, during the occupation time, were calculated and presented in Table 7 and Figure 20. The case that resulted in the maximum number of hours during which the iTs were within the comfort zone range was case No.4, followed by Case No.3, Case No. 1, and Case No.2.

**Table 7:** Number of comfort hours during occupation hours for the B.C and the simulated techniques; August.

August comfort hours (90% acceptable limits; ASHRAE 55–2017)						
Day	B.C-Open	B.C-Closed	Case 1	Case 2	Case 3	Case 4
1-Aug	2	0	2	3	7	4
2-Aug	3	1	3	2	6	3
3-Aug	2	1	3	3	3	8
4-Aug	1	1	3	3	5	10
5-Aug	1	1	3	3	5	3
6-Aug	2	1	4	2	7	5
7-Aug	2	1	4	3	7	7
8-Aug	2	1	3	2	4	3
9-Aug	2	1	3	2	4	6
10-Aug	1	0	2	3	4	3
11-Aug	0	0	1	2	3	3
12-Aug	1	0	1	2	4	3
13-Aug	2	1	2	2	6	6
14-Aug	2	0	2	2	4	3
15-Aug	3	0	2	2	3	9
16-Aug	1	0	2	2	10	9
17-Aug	0	0	1	1	4	5
18-Aug	0	0	1	0	4	4
19-Aug	0	0	0	0	3	3
20-Aug	2	0	1	1	4	5
21-Aug	1	0	1	2	10	10
22-Aug	1	0	2	2	9	9
23-Aug	0	0	2	2	10	9
24-Aug	1	0	2	2	8	8
25-Aug	1	0	1	1	4	5
26-Aug	1	0	1	2	6	5
27-Aug	1	0	2	2	5	5
28-Aug	1	0	1	1	7	6
29-Aug	0	0	1	1	10	10
30-Aug	0	0	1	1	10	10
31-Aug	0	0	1	1	3	4
No. comfort Hours	36	9	58	57	179	183
Total Hours = 310 (8:00 AM - 6:00 PM)	12%	3%	19%	18%	58%	59%

During August, the iTs for the B.C, during the occupation period, were mostly above the acceptable limits; thus the comfort hours were very low. The simulated systems were able to introduce more outdoor air to the classroom, as mentioned in the previous section. In general, the outdoor DB temperatures were within or below the comfort range. Unlike the heating season, the addition of more air to the indoor space resulted in a significant increase in the number of hours during which the iTs fall into the comfort range.

The comparison of Case No.1 to Case No.2, and Case No.3 to Case No.4 indicated that opening the windows did not have a significant effect on increasing the number of comfort hours during the occupation time. Opening the windows reduced the iTs outside the occupation hours, making the night cooling ventilation more effective.

It was found that:

• In February, the heating season, the percentage of comfort hours in the B.C are limited: 16% of the total occupation hours when the windows are closed and 26% when the windows are open. In addition, during the cooling season, August, the percentage of the comfort hours in the B.C is 3% and 12% from the total occupation hours, when the windows are closed and when the windows are open, respectively. This calls for having cooling and heating strategies to increase the number of comfort hours to make the classroom more thermally comfortable.
• In the heating season, the introduction of low DB temperature air to the space would help to increase the number of comfort hours. However, increasing the amount of added air would decrease the iTs below the comfort range, thus reducing the number of comfort hours.
• In the cooling season, the number of comfort hours will be increased when more low DB outdoor air is added to the space. However, as the outdoor air becomes colder during the night, outside of the occupation hours, the comfort hours would not be significantly increased during the occupation hours. However, this may help in nocturnal convection cooling if the building is designed and constructed for this strategy.
• The simulation results of the proposed passive techniques could significantly increase the number of comfort hours. The implementation of these suggested techniques would help to reduce the number of operation hours of the HVAC system, thus saving energy and reducing the running cost.
• Natural ventilation systems and strategies could increase the number of comfort hours in hot arid areas.
• Because opening the windows creates a noise issue, Case No.1 and Case No.3 are feasible to be implemented in this case. Case No.1 would result in a dramatic increase in the percentage of comfort hours in January; from 16% to 57%. However, the improvement of the percentage of comfort hours during August would only increase from 3 to 19%. Case No.3 would result in a significant improvement in the percentage of the comfort hours during February and August; 16%–54%, and 3%–58%, respectively. Case No.3 is the recommended strategy that should be implemented.

### 3.2 Ventilation rate (VR)

**Figure 21:**
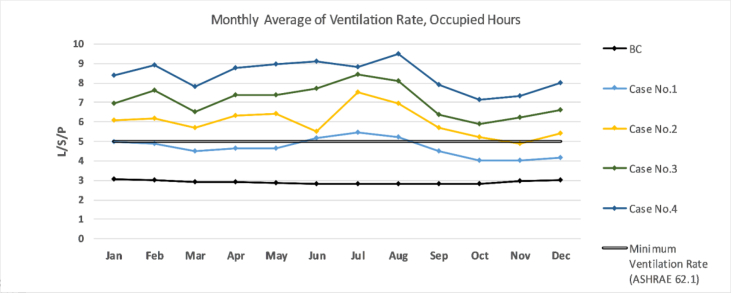
Average Monthly VR for all Cases during the occupation hours.

The annual comparison of the monthly average VR in the B.C and after simulation of the suggested retrofitting modifications is shown in Figure 21. According to the figure, the average VR was not sufficient in the B.C and in Case No.1 in all months, as it was below ASHRAE standard 62–2019; 4.3 L/S/P. In other cases, the average monthly VR would increase in all months. The wind tower will harness and circulate the air through space, thus the amount of air entering the space will increase. Also, when the solar chimneys extract the air from the interior it would allow more air to enter the space through the wind tower. Increasing the area of the operable windows would increase the amount of air entering the space.

Increasing the VR inside the space needs further investigation with detailed computational fluid dynamics (CFD) analysis to study the introduced air inside the space in terms of time, air behavior inside the space, and air velocity. According to ASHRAE 55–2017, in spaces where occupants are without control over the local air speed, the average air speed should stay with the following limits:

a. Operative temperatures above 25.5 °C; the upper limit of the average air speed should not exceed 0.8 m/s.
b. Operative temperatures between 23.0 and 25.5 °C; the upper limit of the average air speed should follow this equation:

(6) Va = 50.49–4.4047 (to) + 0.096425 (to)^2^ (m/s, °C)

in which Va: average air speed, to: Operative temperaturec. Operative temperatures below 23.0 °C; the limit of the average air speed should not exceed 0.2 m/s.

It was found that:

• The monthly average of VR in B.C was between 2–3.5 L/s per person, which is below the recommended rate of ASHRAE standard 62.1–2019.
• The wind tower had a positive effect by increase the air change in the classroom, because it increased the amount of air passing through it.
• When solar chimneys were used to assist the wind tower, the wind tower became more efficient and increased the VR as the solar chimneys helped extract hot air from the space and worked as an exhaust shaft.
• Other parameters related to VR such as airs peed, air behavior inside the space, and corresponding time are needed to get a full picture of the introduced air and the compatibility of the strategies with other comfort standards.

### 3.3 CO_2_ concentration

**Figure 22:**
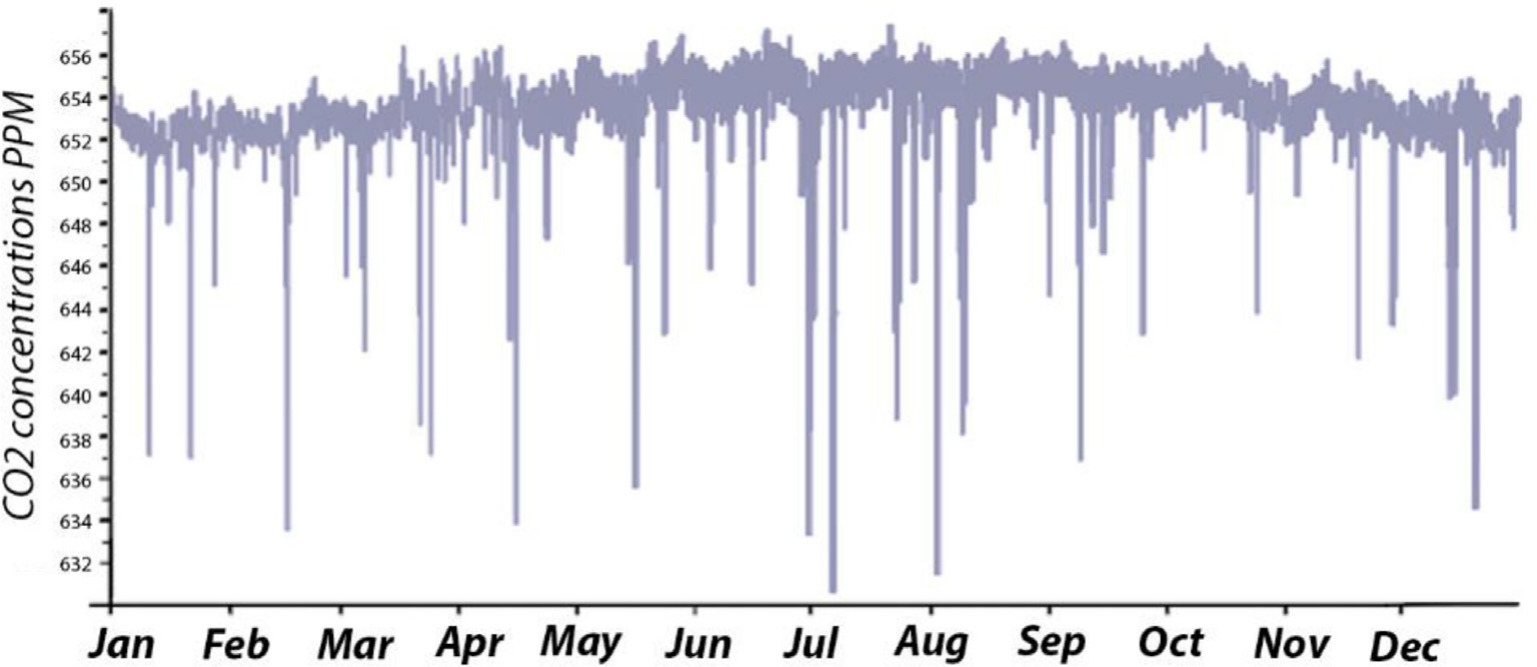
Annual CO_2_ concentration inside the classroom in the B.C (ppm).

**Figure 23:**
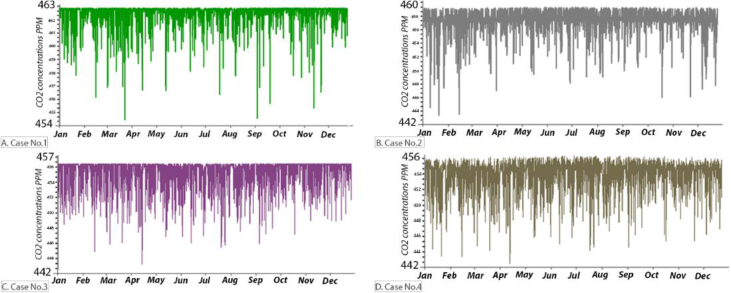
Annual CO_2_ concentration inside the classroom for Case No.1, Case No.2, Case No.3, and Case No.4 (ppm).

The annual CO_2_ concentration for the B.C and after simulating the suggested retrofitting modifications are presented in Figures 22 and 23. In all cases, the CO_2_ concentration was below the maximum limit determined by ASHRAE standard 62, as the monthly average outdoor CO_2_ concentration obtained from the local authority was in the range between 350-450 ppm. Also, the monthly CO_2_ concentration was below the set point recommended by REHVA (1500 ppm) and CIBSE (1000 ppm). Increasing the VR and having more fresh air helped to decrease the maximum CO_2_ concentration (658 ppm in the B.C) by approximately 195 ppm, 201 ppm, 201 ppm, and 202 ppm in Case No. 1, 2, 3, and 4, respectively.

It was found that:

• The values of the CO_2_ concentration were within the acceptable range of ASHRAE standard 62–2019, REHVA, and CIBSE Application Manual AM10 for all cases.
• The modifications resulted in a decrease in the CO_2_ concentration. This decrease was owing to the increase of the VR and more fresh air entering the classroom.

### 3.4 Relative humidity (RH)

**Figure 24:**
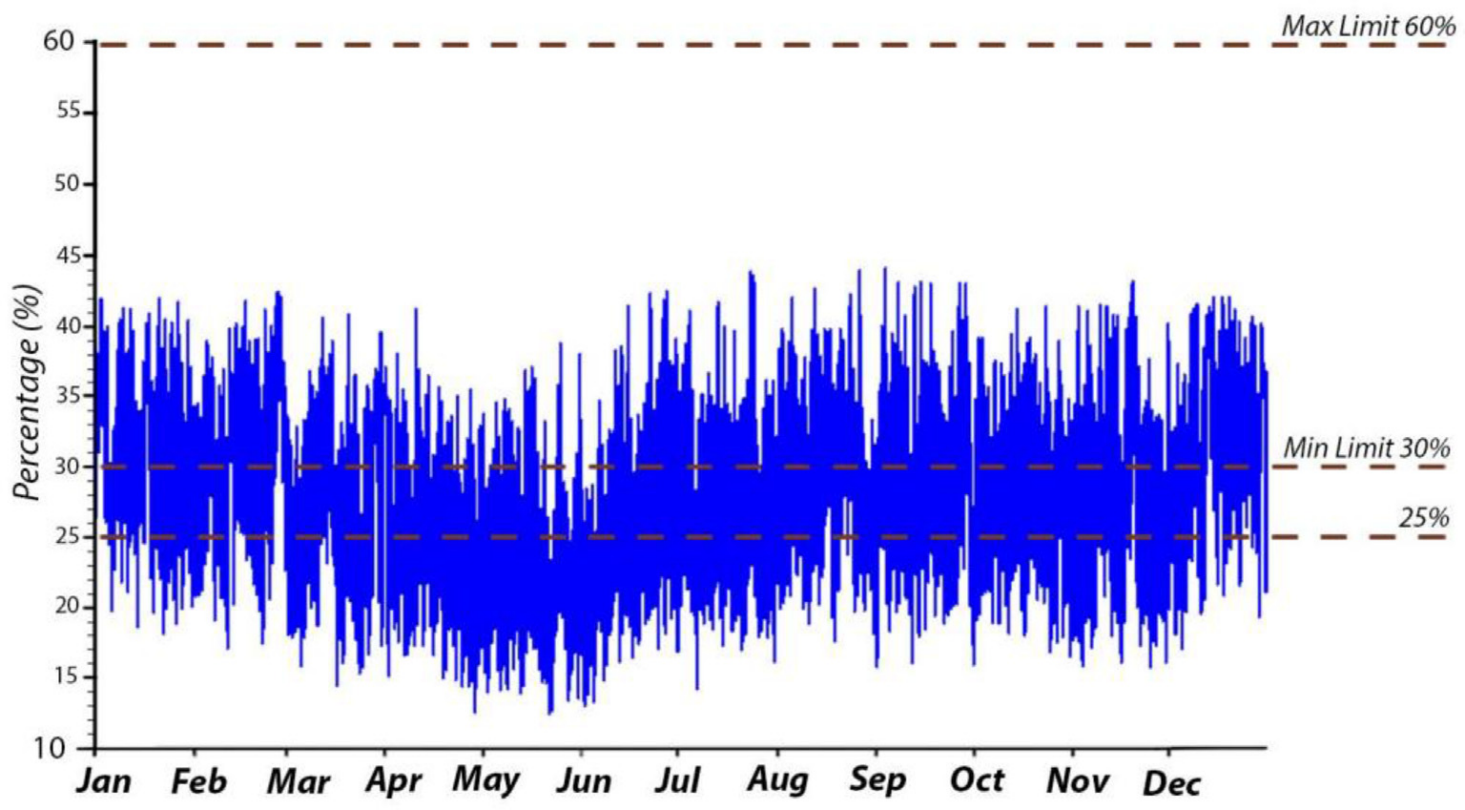
Annual RH inside the classroom in the B.C.

**Figure 25:**
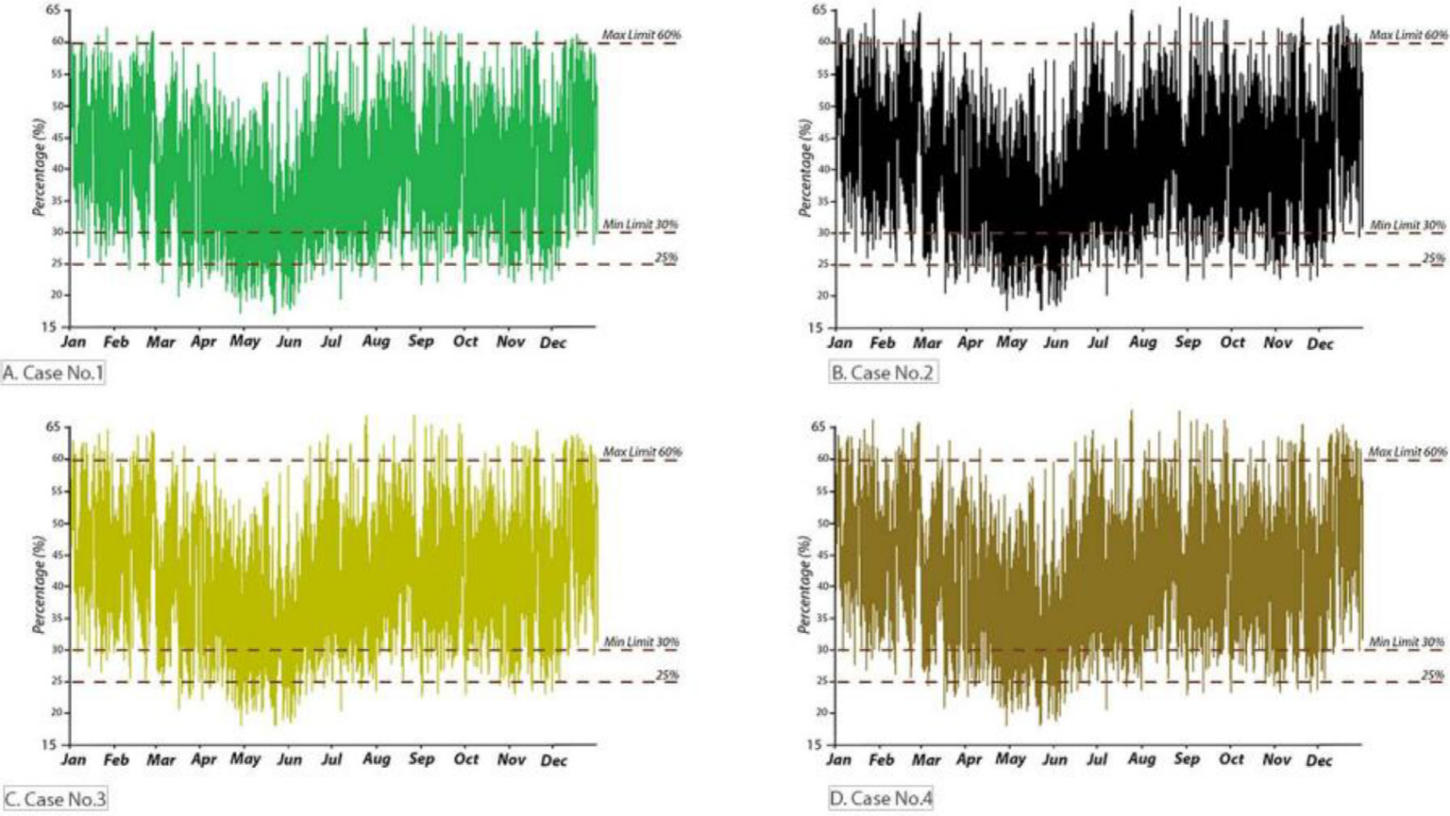
Annual RH inside the classroom for Case No.1, Case No.2, Case No.3, and Case No.4.

The annual RH for the B.C and after simulating the suggested retrofitting modifications are presented in Figures 24 and 25. In all cases, the RH was mostly below the recommended limits determined by ASHRAE standard 62; 65%). If the RH in the B.C was below 30%, especially in the summer months, the proposed strategies help to increase the VR and more fresh air, which helps to raise the RH. Increasing the RH will help to avoid any possible discomfort related to skin drying, irritation of mucus membranes, dryness of the eyes, and static electricity generation.

### 3.5 Energy analysis

The simulation results show that the ventilation strategy described in Case No.3 would enhance the IAQ in terms of iT, VR, RH, and CO_2_ concentration. To examine the energy-saving potentials if this strategy is implemented, energy simulation was performed for the B.C, and for the suggested strategy, and results are as shown in Table 8.

**Table 8:** Annual heating and cooling load energy consumption for B.C and Case No.3

Month	B.C – Heating and cooling Energy consumption (KW)	Case No.3 – Heating and cooling energy consumption (KW)	Saving %
January	1627.5	830.0	49%
February	1463.0	790.0	46%
March	1612.5	935.2	42%
April	1674.0	1054.6	37%
May	1650.0	1155.0	30%
June	1720.5	1101.1	36%
July	2162.2	1340.5	38%
August	1680.0	974.4	42%
September	1680.0	957.6	43%
October	1635.0	1046.4	36%
November	1658.5	1127.7	32%
December	1635.2	948.4	42%
Total	20,198.5	12,261.2	39%
For 152 classrooms	3,070,172.0	1,863,710.0	39%

As shown in Table 8, the adaptation of Case No.3 would save 7,937.3 KW heating and cooling energy annually. If this strategy is implemented in all JUST classroom (162 classes), that would save 1,206,462 KW, which represent 39% of the total cooling and heating energy consumed by the installed HVAC systems.

### 3.6 Economic analysis

The cost of electricity at JUST is based on the tariffs set by national electrical power company (NEPCO) summarized in Table 9.

**Table 9:** Electricity tariff for an educational institute in Jordan.

Monthly electricity consumption	Tariff (JD/kWh)
First Block: from 1-160 kWh/Month	0.042
Second Block: from 161-300 kWh/Month	0.092
Third Block: from 301-500 kWh/Month	0.109
Fourth Block: from 501-600 kWh/Month	0.145
Fifth Block: from 601-750 kWh/Month	0.169
Sixth Block: from 751-1000 kWh/Month	0.190
Seventh Block: more than 1000 kWh/Month	0.256

The cost of the yearly energy savings was calculated according to the NPECO tariff, as shown in Table 10. The energy price was assumed to be constant during the lifetime of the system. The net cash flows were calculated yearly and assumed to be constant over the system's lifetime.

**Table 10:** Monthly energy consumption and cost of heating and cooling loads and the cost of energy-saving.

Month	B.C – Heating and cooling energy consumption (kWh)	Case No.3 – Heating and cooling energy consumption (kWh)	Monthly Saving in kWh	Monthly cost of energy-saving (JD)
January	1627.5	830.0	797.5	192.9
February	1463.0	790.0	673.0	158.4
March	1612.5	935.2	677.3	169.1
April	1674.0	1054.6	619.4	158.8
May	1650.0	1155.0	495.0	126.9
June	1720.5	1101.1	619.4	158.8
July	2162.2	1340.5	821.7	210.6
August	1680.0	974.4	705.6	178.9
September	1680.0	957.6	722.4	182.1
October	1635.0	1046.4	588.6	150.9
November	1658.5	1127.7	530.8	136.1
December	1635.2	948.4	686.8	172.2
Total				1996.1

The initial cost of the proposed system (Case No.3), including the cost of materials and installation) was estimated based on an average quote from three different local contractors, which was 2,100 JD for one classroom. The annual operation and maintenance cost was estimated to be 2% of the total initial cost. The discount rate was 3.5% based on the central bank of Jordan. The system lifetime was assumed to be 25 years. The cost-effectiveness of implementing case No.3 in one classroom is summarized in Table 11.

**Table 11:** Cost-effectiveness of implementing the proposed system as described in Case No.3

Item	Unit	Value
The initial cost of the proposed system	JD	2100
Operation and maintenance cost	JD	42
Discount Rate	%/100	3.5
System lifetime	Years	25
Cost of yearly energy saving (AES∗Pr)	JD	1996.1
SPP	Years	1.05
NPV	JD	30795.72
PVS	JD	32895.72
UPVF	---	16.48
B/C		15.66

The evaluation of the economic life cycle assessment is necessary to determine the economic viability of the suggested system (Case No.3). The results in Table 11 shows:

• The simple payback period is less than the life of the proposed system.
• The present value of energy-saving is greater than the cost of the initial investment, and the net present value is 30795.75 JD, which means a positive investment indicator.
• The benefit-cost ratio is 15.66, which is another indicator of positive investment in implementing the suggested system.

The electricity cost is one of the financial burdens at JUST. Jordan imports approximately 97% of its primary energy, which makes the energy situation very critical. The continuous growth in energy demand requires urgent actions towards the adoption of energy-saving strategies, as suggested in this research, in addition to other benefits indicated previously. The implementation of the proposed system will provide other environmental benefits such as lower CO_2_ emission and mitigating air pollution.

## 4 Conclusions

Different natural ventilation retrofitting techniques to improve the IAQ of existing classrooms were investigated through simulation scenarios. A classroom located at JUST was used as a model to evaluate the performance of the suggested techniques through computer simulations. The proposed retrofitting techniques were designed by taking into account the special construction and architectural design of JUST with minimal impact and disruption of the building envelope. Four cases were proposed and evaluated using computer simulations. The simulations results were used to assess the performance of each Case.

The IAQ performance was evaluated in terms of *iTs*, VR, and CO_2_ levels. The natural ventilation retrofitting techniques were evaluated over a year during the usual occupied hours (8.00 AM- 6.00 PM), taking the month of February as an example of Winter seasons, and the month of August as an example of Summer seasons. The months were selected to coincide with the JUST annual teaching calendar.

A positive effect on the IAQ was observed in all four cases. However, Case No.2 and Case No.4 were simulated with the windows open, thus they were excluded owing to the noise issue from the adjacent courtyards. The number of comfort hours during August were slightly increased in Case No.1. The results obtained with a wind tower assisted with a solar chimney, Case No.3, were positive in August and February, the number of comfort hours increased by approximately 106 h during February, and 170 h during August.

The average monthly VR in the B.C was always below the recommended rate of ASHRAE standard 62.1–2019; 2–3.5 L/S/P. However, in Case No.3 the VR in all months was always above the recommended rate; 4.3 L/S/P. More investigations are needed to correlate the VR with the hours during which the iTs are within the comfort zone. In all cases, the CO_2_ concentrations were within the acceptable range of ASHRAE standard 62–2016, REHVA, and CIBSE. This was expected as the outdoor CO_2_ concentration was between 350-400 ppm, and introducing more outdoor air would decrease the CO_2_ concentration inside the space. In the B.C, RH values were mainly below the recommended limits, 30%, whereas the simulated strategies increased the RH values; they were still within the upper limits recommended by ASHRAE 62–2016.

Adapting the natural ventilation strategy described in Case No.3, wind tower with solar chimney, as a natural ventilation retrofit measure in all JUST classrooms would reduce the heating and cooling energy consumption to 1,863,710.0 KW, which represents a 39% saving compared with the use of split unit HVAC systems. An experimental study is needed to validate the simulation results of Case No.3. An economic assessment of the suggested system indicates positive NPV and B/C ratio values, which indicate that the proposed ventilation system is a viable energy-saving system in addition to the other described benefits.

For future research, the authors suggest having an experimental design and implementing the proposed techniques to validate the simulation results and confirm the IAQ results. The indoor airspeed and noise penetration should also be investigated.

## Declarations

### Author contribution statement

Shouib Nouh Mabdeh & Amani Al-Zghoul: Conceived and designed the experiments; Performed the experiments; Analyzed and interpreted the data; Contributed reagents, materials, analysis tools or data; Wrote the paper.

Tamer Alradaideh: Performed the experiments; Analyzed and interpreted the data; Contributed reagents, materials, analysis tools or data; Wrote the paper.

Asma Bataineh & Saba Ahmad: Performed the experiments; Analyzed and interpreted the data; Wrote the paper.

### Funding statement

This research did not receive any specific grant from funding agencies in the public, commercial, or not-for-profit sectors.

### Competing interest statement

The authors declare no conflict of interest.

### Additional information

No additional information is available for this paper.
